# Intravenous Carbetocin Versus Rectal Misoprostol for the Active Management of the Third Stage of Labor: A Systematic Review and Meta-Analysis of Randomized Controlled Trials

**DOI:** 10.7759/cureus.30229

**Published:** 2022-10-12

**Authors:** Ebraheem Albazee, Hanaa Alrashidi, Roa Laqwer, Shouq R Elmokid, Wessam A Alghamdi, Hend Almahmood, Muneera AlGhareeb, Nora Alfertaj, Danah I Alkandari, Fatma AlDabbous, Jaber Alkanderi, Haifa Al-Jundy, Ahmed Abu-Zaid, Osama Alomar

**Affiliations:** 1 Department of Internship, Kuwait Institute for Medical Specializations, Kuwait City, KWT; 2 College of Medicine, Alfaisal University, Riyadh, SAU; 3 College of Medicine and Medical Science, Arabian Gulf University, Manama, BHR; 4 Department of Obstetrics and Gynecology, Dr. Sulaiman Al-Habib Hospital, Riyadh, SAU; 5 College of Graduate Health Sciences, The University of Tennessee Health Science Center, Memphis, USA; 6 Department of Obstetrics and Gynecology, King Faisal Specialist Hospital and Research Center, Riyadh, SAU

**Keywords:** vaginal birth, meta-analysis, systematic review, postpartum hemorrhage, misoprostol, carbetocin

## Abstract

Globally, postpartum hemorrhage (PPH) is the top cause of maternal death. Multiple uterotonic medications are available to prevent PPH; however, it is still unclear whether one is the most effective. The current study compared the efficacy and safety of intravenous carbetocin with rectal misoprostol for the active management of the third stage of labor in order to prevent PPH. Eligible studies were found utilizing digital medical sources, including the Cochrane Central Register of Controlled Trials (CENTRAL), Web of Science (WOS), PubMed, Scopus, and Google Scholar, from inception until September 2022. Only randomized controlled trials (RCTs) that matched the inclusion requirements were chosen. We used the Cochrane Risk of Bias scale (version 2) to assess the quality of the included studies. The Review Manager (version 5.4 for Windows) was used to conduct the meta-analysis. The results were summarized as mean difference (MD) or risk ratio (RR) with a 95% confidence interval (CI) in fixed- or random-effects models according to the degree of between-study heterogeneity. Collectively, we screened 621 articles after omitting duplicates and eventually included three RCTs for analysis. Overall, 404 patients were included in these studies; 202 patients were allocated to the intravenous carbetocin group whereas 202 patients were allocated to the rectal misoprostol group. Two RCTs were judged as “low” risk of bias, whereas one RCT was judged as having “some concerns” regarding the quality assessment. Regarding efficacy endpoints, the intravenous carbetocin group had significantly lower blood loss (n=3 RCTs, MD=-117.74 mL, 95% CI [-185.41, -50.07], p<0.001), need for additional uterotonics (n=2 RCTs, RR=0.06, 95% CI [0.01, 0.46], p=0.007), need for uterine massage (n=2 RCTs, RR=0.40, 95% CI [0.20, 0.80], p=0.009), and need for blood transfusion (n=2 RCTs, RR=0.38, 95% CI [0.15, 0.95], p=0.04) compared with the rectal misoprostol group. Regarding safety endpoints, the rates of diarrhea (n=3 RCTs, RR=0.18, 95% CI [0.06, 0.55], p=0.003) and chills (n=2 RCTs, RR=0.31, 95% CI [0.12, 0.83], p=0.02) were significantly lower in the intravenous carbetocin group compared with the rectal misoprostol group. However, there was no significant difference between both groups regarding the rates of headache (n=3 RCTs, RR=1.23, 95% CI [0.06, 1.91], p=0.35) and facial flushing (n=2 RCTs, RR=0.88, 95% CI [0.46, 1.68], p=0.70). In conclusion, it was discovered that intravenous carbetocin was a superior substitute for rectal misoprostol for the active management of the third stage of labor. With far fewer side effects, intravenous carbetocin decreased postpartum blood loss and further uterotonic use. For women who have a high risk of PPH, intravenous carbetocin is advised.

## Introduction and background

According to a global estimation in 2015, approximately 303,000 women lost their lives while giving delivery [1]. Up to one-third of these maternal deaths were caused by postpartum hemorrhage (PPH) [2]. The majority of deaths happened in developing or middle-income nations [3,4]. According to a comprehensive analysis, the global prevalence of PPH is estimated to be 10.8% [5]. However, there is significant regional variability, with rates ranging from 7.2% in Oceania to reach the maximum level of 25.7% in Africa [5].

The diagnosis of PPH is confirmed when the blood loss from the female reproductive tract exceeds 500 mL within the first day following a vaginal delivery or exceeds 1,000 mL following a cesarean section [6]. In about 70% of cases, uterine atony, which is described as the inability of the uterus to contract after giving birth, is the root cause of PPH [7]. As a result, the World Health Organization (WHO) supports active management of the third stage of labor and the administration of uterotonic medications as prevention against PPH in all females giving birth [8]. However, despite the administration of prophylactic medications, some investigations have shown that 6% to 16% of women still experience blood loss exceeding 500 mL [9].

The best method for treating and preventing PPH is by administering oxytocin [10]. The primary function of the peptide hormone oxytocin, released by the posterior pituitary, is to increase uterine contractions during childbirth and prevent PPH [11,12]. On the other hand, the direct intravenous injection of oxytocin may result in discomfort, diarrhea, seizures, and hypervolemia [13]. Additionally, it is both photo- and thermo-labile and requires some precautions such as sterilization [14]. Numerous alternative medications have been investigated over the past 20 years to overcome oxytocin drawbacks, including prostaglandins such as misoprostol [15] or oxytocin analogs such as carbetocin [16].

Carbetocin, an oxytocin analog with a long half-life, attaches to the receptors on the uterine muscle fibers, causing uterine contractility and enhancing the amplitude of the current uterine contractions and uterine tone [17]. Compared with oxytocin, it has been linked to a considerable decline in the prevalence of PPH following cesarean section, as well as a reduced requirement for other uterotonic medications and uterine massaging following natural birth [16].

Misoprostol is an artificial analog of prostaglandins, and it works by activating the G proteins that usually trigger adenylate cyclase. It is beneficial in preventing and treating PPH because it increases the frequency and intensity of uterine contractility during pregnancy [18]. It is affordable and thermally stable; thus, unlike oxytocin, it does not need to be refrigerated. It can be administered in various ways, including orally, rectally, and vaginally. However, it has been demonstrated that rectally delivered misoprostol has a lower rate of side effects than misoprostol taken orally in cases of bleeding [14].

The critical job of a gynecologist is to prevent PPH with as few adverse effects as possible. To the best of our knowledge, no meta-analysis has previously looked into the clinical utility of intravenous carbetocin versus rectal misoprostol for the prevention of PPH among patients who underwent vaginal delivery. Therefore, the current study compared the efficacy and safety of intravenous carbetocin with rectal misoprostol for the active management of the third stage of labor in order to prevent PPH.

## Review

Methods

Data Sources and Search Strategy

The study protocol was not retrospectively recorded in the International Prospective Register of Systematic Reviews (PROSPERO). This investigation followed the instructions outlined in the Cochrane Handbook for Systematic Reviews of Interventions [19], as well as the Preferred Reporting Items for Systematic Reviews and Meta-Analyses (PRISMA) declaration [20]. Being a systematic review and meta-analysis, our study did not require an official ethical clearance.

We looked through digital medical sources, including the Cochrane Central Register of Controlled Trials (CENTRAL), Web of Science (WOS), PubMed, Scopus, and Google Scholar, from inception until September 2022. For our search, we applied the following search strategy: (“normal delivery” OR “vaginal delivery” OR “vaginal birth”) AND (carbetocin OR pabal OR depotocin OR duratocin OR lonactene) AND (misoprostol OR “novo misoprostol” OR “apo misoprostol” OR cytotec OR “SC30249” OR “SC29333” OR glefos OR misodel OR mysodelle OR misotac). In order to expand the literature review, we also looked at the reference lists of the articles we had collected. During our search, we also considered clinicaltrials.gov and the clinical trial registry of the WHO. The literature search was not limited by the date of publication, country, or language.

Study Selection

Our inclusion criteria comprised: (i) patients: women undergoing vaginal delivery; (ii) intervention: intravenous carbetocin; (iii) comparator: rectal misoprostol; (iv) outcomes: select efficacy and safety endpoints; and (v) study design: human-based randomized clinical trials (RCTs). We excluded all other study designs other than RCTs or procedures other than vaginal delivery. Also, we did not include research whose extracted data were unreliable for analysis.

Separately, two authors checked the titles and abstracts of all relevant studies in the sources, deleted duplicates, and determined eligibility by full-text screening. Additionally, the references of the final included papers were manually reviewed to find missing or additional citations. Discussions were used to settle disagreements.

Data Extraction and Quality Assessment of Studies

We used the Cochrane Risk of Bias scale (version 2) [21] to assess the quality of the included studies. This assessment was done by two authors separately. The following elements are examined by this tool: the randomization process, deviation from the intended interventions, missing outcome data, outcome measurement, and selection of the reported result. The authors assigned a risk level of low, unclear, or high to each tool domain and the general quality of the selected papers. Discussions were used to settle disputes. Publication bias is unreliable for pooled studies with fewer than ten trials [22]. Hence, Egger's test for funnel plot asymmetry could not be used in our analysis to determine whether there was a publication bias.

Two authors separately extracted the data using a standard form. Discussions were used to settle conflicts. The primary three categories of data were collected. First, we compiled a list of the attributes of the studies that were included, such as a trial identifier, nation, length of the trial, sample size, and study arms. Second, we gathered information on the patient's baseline characteristics, including sample size, age (years), gestational age (weeks), parity, body mass index (BMI), and birth method. Third, we gathered information on efficacy outcomes such as blood loss (ml), transfusion need, uterine massage need, and further uterotonics need. Additionally, we gathered information on safety outcomes such as headache, diarrhea, nausea, vomiting, chills, and facial flushing.

Statistical Analysis

The Cochrane Collaboration's Review Manager Program was used to analyze the data. In order to calculate the risk ratio (RR) and mean difference (MD) with a 95% confidence interval (Cl), respectively, we combined the dichotomous and continuous data. The Inverse-Variance and Mantel-Haenszel techniques were used, respectively, for the analyses. By looking at the graphs on the forest plots, heterogeneity was evaluated, and the degree of heterogeneity was determined using the chi-square and I-square (I^2^) tests. Significant heterogeneity was determined to be chi-square test with p<0.1 and I^2^ test >50% [23]. Fixed- and random-effects models were used to assess the homogeneous and heterogeneous outcomes. Leave-one-out sensitivity analysis was done to resolve the heterogeneous outcomes, if available, by omitting one study at a time and re-pooling the effect size of the remaining studies. If the endpoints had a p-value of 0.05 or lower, we considered them statistically significant.

Results

Literature Search Results

**Figure 1:**
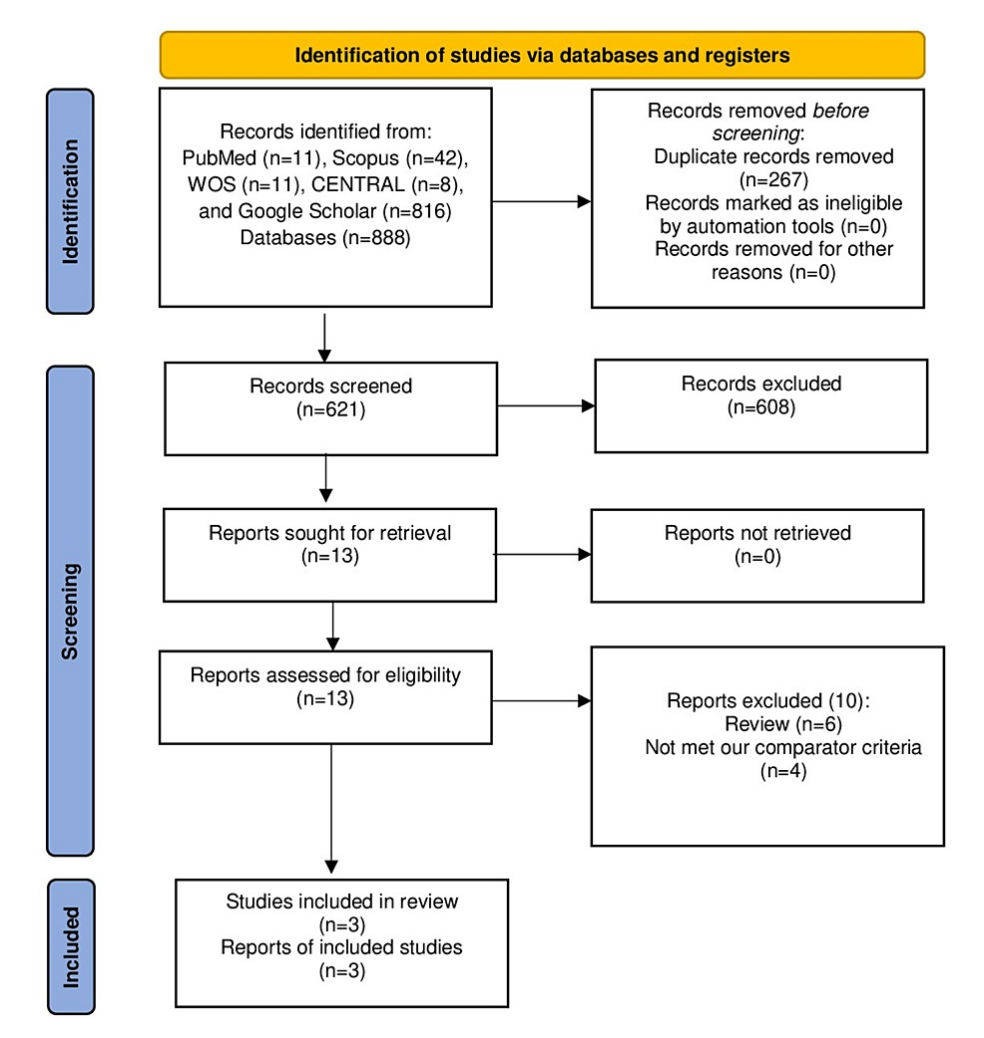
The Preferred Reporting Items for Systematic Reviews and Meta-Analyses (PRISMA) flow diagram.

Our search yielded 621 citations after removing 267 duplicates. After that, 608 citations were excluded during title/abstract screening. Finally, three RCTs [24-26] met our criteria after excluding ten studies during full-text screening. Figure 1 depicts the PRISMA flow diagram for our screening process. Overall, 404 patients were included in these studies; 202 patients were allocated to the intravenous carbetocin group whereas 202 patients were allocated to the rectal misoprostol group.

Study Characteristics

All included RCTs were from Egypt but with different durations and trial settings. All included RCTs had the same intravenous carbetocin dose. However, each study had different rectal misoprostol doses. Tables 1, 2 depict a summary of the baseline characteristics of the included trials and participants, respectively.

**Table 1:** Summary of the baseline characteristics of the included trials.

Study identifier	Country	Trial duration, (hospital)	Total sample size, n	Study arms	
				Intervention	Control
Maged 2019 [26]	Egypt	Between July 2018 and May 2019, (Kasr Al Ainy)	n=150	Carbetocin 100 μg/mL (IV)	Two misoprostol tablets 800 μg (rectal)
Abd El-Wahab 2020 [24]	Egypt	Between March 2019 and August 2019, (Beni Suef)	n=160	Carbetocin 100 μg/mL (IV)	Four misoprostol tablets 800 μg (rectal)
Hetiba 2021 [25]	Egypt	Between December 2019 and December 2020, (Al-Azhar)	n=94	Carbetocin 100 μg/mL (IV)	Three misoprostol tablets 600 μg (rectal)

**Table 2:** Summary of the baseline characteristics of the included participants.

Study ID	Group	Sample size, n	Age (years)	Gestational age (weeks)	Parity	BMI (kg/m²)	Type of delivery	
								Maged 2019 [26]	Carbetocin	n=75	26 ± 4.2	38.2 ± 0.9	1 ± 0.66	29.9 ± 1.2	Vaginal	
Misoprostol	n=75	27.3 ± 6.4	38 ± 1	1 ± 0.66	29 ± 1.3										
Abd El-Wahab 2020 [24]	Carbetocin	n=80	28.2 ± 4.26	37.8 ± 1.26	2.04 ± 1.09	Not available	Vaginal	
	Misoprostol	n=80	29 ± 3.81	38.2 ± 1.17	1.84 ± 0.96			
Hetiba 2021 [25]	Carbetocin	n=47	30.02 ± 7.68	Not available	2.13 ± 1.78	30.59 ± 4.6	Vaginal	
	Misoprostol	n=47						

Quality Assessment

**Figure 2:**
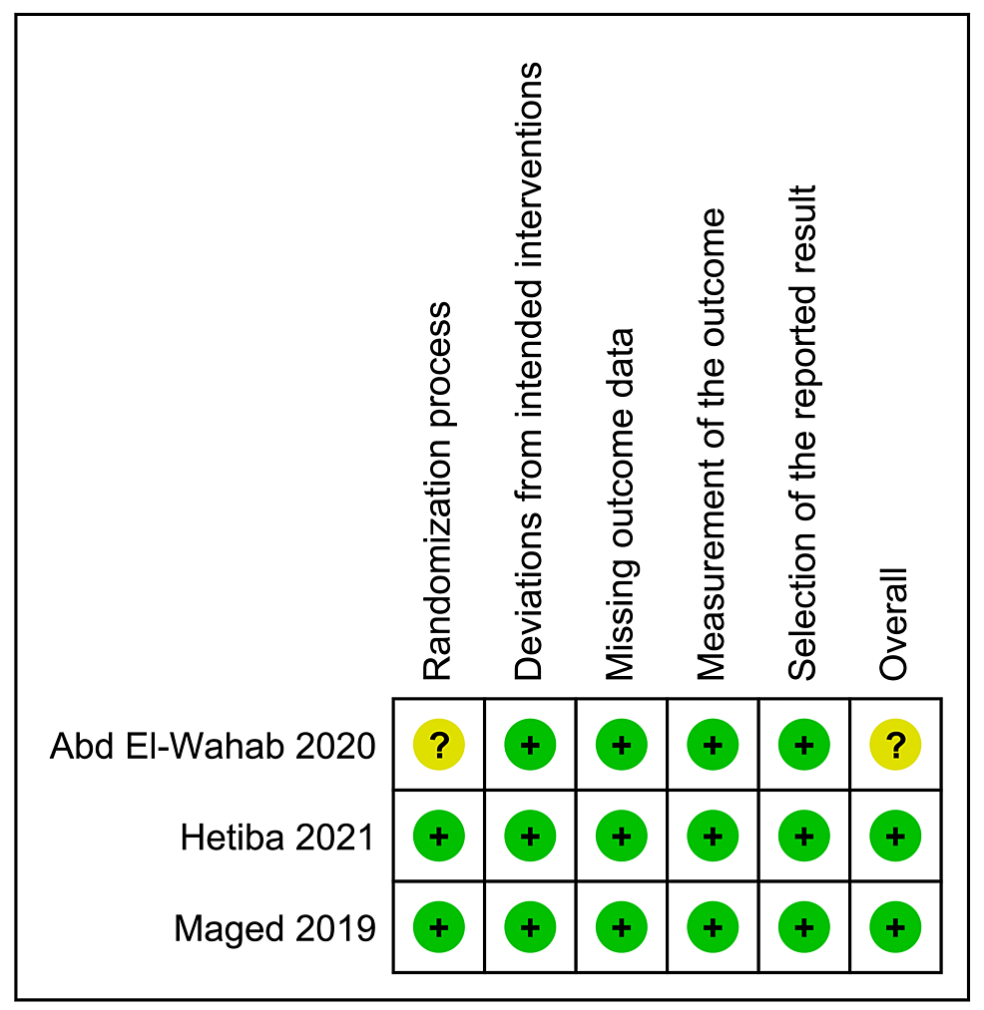
Summary of the risk of bias in the included trials. ?: unclear risk of bias, +: low risk of bias. Cited articles: [24-26].

Figure 2 depicts the quality assessment of the included RCTs. Two RCTs [25, 26] were evaluated as having a “low” risk of bias. However, one study [24] was assessed as having an “unclear” risk of bias, because it provided no information about the randomization process.

Results of the Meta-Analysis

**Figure 3:**
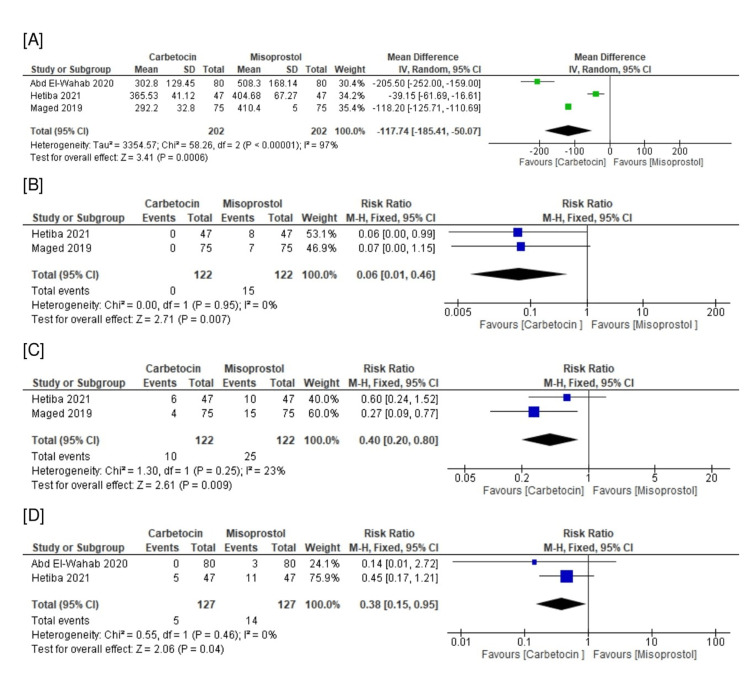
Meta-analysis of the efficacy endpoints: (A) blood loss (mL), (B) need for additional uterotonics, (C) need for uterine massage, and (D) need for blood transfusion. Cited articles: [24-26].

Regarding efficacy endpoints, the carbetocin group had significantly lower blood loss (n=3 RCTs, MD=-117.74 ml, 95% CI [-185.41, -50.07], p<0.001), need for additional uterotonics (n=2 RCTs, RR=0.06, 95% CI [0.01, 0.46], p=0.007), need for uterine massage (n=2 RCTs, RR=0.40, 95% CI [0.20, 0.80], p=0.009), and need for blood transfusion (n=2 RCTs, RR=0.38, 95% CI [0.15, 0.95], p=0.04) compared with the rectal misoprostol group. All the pooled analyses were homogeneous (chi-square p>0.1 and I^2^<50%), except for blood loss (chi-square p<0.001 and I^2^=97%) (Figures 3A-3D). The heterogeneous outcome of postpartum blood loss could not be resolved by leave-one-out sensitivity analysis.

**Figure 4:**
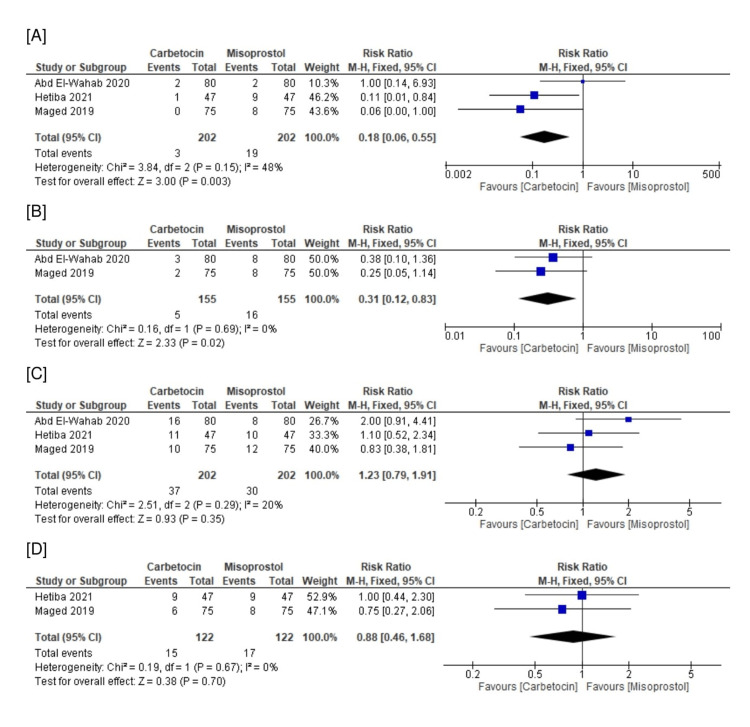
Meta-analysis of the rate of safety endpoints: (A) diarrhea, (B) chills, (C) headache, and (D) facial flushing. Cited articles: [24-26].

Regarding safety endpoints, the rates of diarrhea (n=3 RCTs, RR=0.18, 95% CI [0.06, 0.55], p=0.003) and chills (n=2 RCTs, RR=0.31, 95% CI [0.12, 0.83], p=0.02) were significantly lower in the carbetocin group compared with the rectal misoprostol group. However, there was no significant difference between both groups regarding the rates of headache (n=3 RCTs, RR=1.23, 95% CI [0.06, 1.91], p=0.35) and facial flushing (n=2 RCTs, RR=0.88, 95% CI [0.46, 1.68], p=0.70). All the pooled analyses were homogeneous (chi-square p>0.1 and I^2^<50%) (Figures 4A-4D).

Discussion

The goal of the current study was to compare how rectal misoprostol and intravenous carbetocin prevented PPH. Our investigation discovered that carbetocin was more efficient than rectal misoprostol for managing the third stage of labor. Compared with rectal misoprostol, intravenous carbetocin significantly lowered the need for additional uterotonic medications or uterine massage. Intravenous carbetocin was also associated with less blood loss and reduced need for blood transfusion. Concerning the adverse events, intravenous carbetocin had significantly lower incidences of diarrhea and chills than rectal misoprostol. However, the administration of misoprostol compared with intravenous carbetocin had an insignificant effect on the incidences of headache and facial flushing.

These findings align with Abd El Aziz et al. [27] and Hetiba et al. [25], who found that blood loss was substantially lower in the intravenous carbetocin arm compared with the misoprostol arm among women who gave birth vaginally or via cesarean surgery. Also, when misoprostol, oxytocin, and carbetocin were tested in a study by Mousa et al. [10], the average blood loss was substantially higher in the misoprostol arm than in the carbetocin arm.

Misoprostol was compared with a placebo in a different trial conducted by Sallam and Shady [28] at an Egyptian hospital to determine which drug minimized blood loss and prevented PPH. It was discovered that the misoprostol arm substantially lowered blood loss compared with placebo. Even in other procedures, such as in women undergoing myomectomy, carbetocin administration was linked to various positive clinical outcomes, including decreased operative blood loss and blood transfusion need [12].

Our analysis stated that intravenous carbetocin was associated with a reduced need for blood transfusion than rectal misoprostol. This result was similar to another study that showed that the requirement for blood transfusion varied depending on the treatment, with no patients requiring blood transfusion in the intravenous carbetocin group compared with three cases in the misoprostol group [24]. On the contrary, the results of another investigation indicated that the type of medicine being used had no impact on the requirement for blood transfusion [25]. Also, Maged et al. [26] and Attilakos et al. [29] found that there was no discernible disparity in the incidence of severe PPH or the need for blood transfusion between carbetocin and oxytocin groups.

The type of drug utilized as prophylaxis against PPH in the current study impacted the requirement for uterotonic medications. In cases where carbetocin was administered as prophylaxis, there was no further need for uterotonic medications. This result was in accordance with the findings of Abd El-Wahab et al. [24]; they found that the requirement for extra uterotonic medications was lower in the carbetocin arm than in the rectal misoprostol arm. In an RCT conducted by Ibrahim and Saad [30], it was discovered that misoprostol dramatically increased the requirements for extra uterotonic medications and blood transfusion compared with carbetocin. In a similar context, Larciprete et al. [31] evaluated carbetocin and oxytocin in women who had undergone cesarean birth and found that carbetocin was related to a reduced need for extra uterotonics.

In our study, the side effects produced a variety of findings; some were statistically significant, while others had no impact. Carbetocin had significantly lower incidences of diarrhea and chills than rectal misoprostol. However, the administration of misoprostol compared with carbetocin had an insignificant effect on the incidences of headache and facial flushing. According to another study, there was a significant difference between the misoprostol and carbetocin groups in the patients who experienced side symptoms such as fever, nausea, diarrhea, and abdominal discomfort; the subjects in the carbetocin category were less likely to experience these adverse events following childbirth. On the other hand, it was discovered that there was no statistically significant difference between the groups receiving carbetocin and misoprostol in cases where there were side symptoms such as allergies, facial flushing, and headache [25].

Because of numerous variables related to population research designs and population characteristics, the results of various studies on the side effects of medications used to prevent PPH reveal many discrepancies and are incompatible with one another. For example, Abd El-Aziz et al. [27] reported that misoprostol had a greater heart rate and heat feeling than carbetocin in terms of adverse effects. Additionally, Ibrahim and Saad [30] published the findings on adverse effects, finding that carbetocin was more frequently linked to nausea, vomiting, and headache. At the same time, misoprostol was more frequently linked to shivering and pyrexia.

Generally speaking, carbetocin is more expensive than misoprostol. In low-resource settings, Bradley and colleagues [14] conducted a cost-effectiveness analysis and highlighted the substantial cost-effectiveness of administering rectal misoprostol to prevent PPH among patients undergoing vaginal delivery. This finding was echoed in another cost-effectiveness analysis that compared misoprostol with no uterotonic agent (n=5 studies) [32]. In the United Kingdom, Matthijsse et al. [33] carried out a cost-effectiveness analysis of carbetocin versus oxytocin for the prevention of PPH among patients who delivered vaginally. The results showed that carbetocin was more cost-effective than oxytocin as it correlated with less number of PPH incidences, lower treatment-related expenses, and better antihemorrhagic efficacy [33]. Nonetheless, a cost-effectiveness analysis at the community level from Senegal showed that oral misoprostol was more cost-effective than intramuscular oxytocin ($40 versus $120, respectively) [34]. Unfortunately, none of the meta-analyzed RCTs examined cost-effectiveness between intravenous carbetocin and rectal misoprostol, and this is a critical limitation of these RCTs. Additionally, to our knowledge, there are no cost-effectiveness analyses that directly compared intravenous carbetocin with rectal misoprostol for prevention of PPH during vaginal delivery, and this represents an important focus for future research. Among patients undergoing cesarean delivery, a network meta-analysis of RCTs depicted that carbetocin (not misoprostol) was the most effective uterotonic agent in decreasing postpartum blood loss [35]. Moreover, among patients undergoing vaginal delivery, a network meta-analysis by Gallos et al. [36] showed that carbetocin was more effective and more costly than all other uterotonic agents including misoprostol. Nonetheless, the relative cost-effectiveness data were questionable as the findings were impacted by uncertainty and discrepancy in the data of the adverse events [36].

Strengths and limitations of the study

Our study harbors several strength points that ought to be emphasized. Most remarkably, we carried out the first-ever meta-analysis on the efficacy and safety of intravenous carbetocin versus rectal misoprostol among patients undergoing vaginal delivery. We pooled data from only RCTs to generate high-quality evidence. Besides, all outcomes, except one, were homogenous, hence suggesting that almost all studies produced a similar consistent effect.

Nevertheless, the current study has some drawbacks. The main drawback was the small number of included trials, which restricted us from evaluating publication bias. Another drawback was the participants' short follow-up times, the lack of blinding of some investigators and/or participants, and the different dosing of rectal misoprostol. Additionally, the study protocol of the present research was not retrospectively recorded in PROSPERO, hence reporting bias could not be fully excluded. Lastly, the findings of the study should be cautiously interpreted in view of their potential weak evidence, and this is because some outcomes were pooled from only two RCTs.

## Conclusions

This systematic review and meta-analysis of three RCTs examined the efficacy and safety of intravenous carbetocin compared with rectal misoprostol for the active management of the third stage of labor. The findings revealed that carbetocin correlated with significantly lower blood loss, need for additional uterotonics, need for uterine massage, and need for blood transfusion compared with rectal misoprostol. Additionally, intravenous carbetocin had significantly lower incidences of diarrhea and chills compared with rectal misoprostol. All in all, intravenous carbetocin was a superior substitute for rectal misoprostol for the active management of the third stage of labor. Nevertheless, additional RCTs with larger sample sizes may be needed to validate these conclusions. Future directions may include studies that apply various administration methods, assess multiple doses, and determine the effects of both drugs.

## References

[1] Alkema L, Chou D, Hogan D (2016). Global, regional, and national levels and trends in maternal mortality between 1990 and 2015, with scenario-based projections to 2030: a systematic analysis by the UN Maternal Mortality Estimation Inter-Agency Group. Lancet.

[2] Say L, Chou D, Gemmill A (2014). Global causes of maternal death: a WHO systematic analysis. Lancet Glob Health.

[3] Gallos ID, Papadopoulou A, Man R (2018). Uterotonic agents for preventing postpartum haemorrhage: a network meta-analysis. Cochrane Database Syst Rev.

[4] Penney G, Brace V (2007). Near miss audit in obstetrics. Curr Opin Obstet Gynecol.

[5] Calvert C, Thomas SL, Ronsmans C, Wagner KS, Adler AJ, Filippi V (2012). Identifying regional variation in the prevalence of postpartum haemorrhage: a systematic review and meta-analysis. PLoS One.

[6] McLintock C (2020). Prevention and treatment of postpartum hemorrhage: focus on hematological aspects of management. Hematology Am Soc Hematol Educ Program.

[7] Oladapo OT, Fawole B, Blum J, Abalos E (2012). Advance misoprostol distribution for preventing and treating postpartum haemorrhage. Cochrane Database Syst Rev.

[8] de Castro Parreira MV, Gomes NC (2013). Preventing postpartum haemorrhage: active management of the third stage of labour. J Clin Nurs.

[9] Mobeen N, Durocher J, Zuberi N (2011). Administration of misoprostol by trained traditional birth attendants to prevent postpartum haemorrhage in homebirths in Pakistan: a randomised placebo-controlled trial. BJOG.

[10] Mousa HA, Blum J, Abou El Senoun G, Shakur H, Alfirevic Z (2014). Treatment for primary postpartum haemorrhage. Cochrane Database Syst Rev.

[11] Clark SL, Simpson KR, Knox GE, Garite TJ (2009). Oxytocin: new perspectives on an old drug. Am J Obstet Gynecol.

[12] Albazee E, Sayad R, Elrashedy AA, Samy Z, Faraag E, Baradwan S, Samy A (2022). Efficacy of oxytocics on reducing intraoperative blood loss during abdominal myomectomy: a systematic review and meta-analysis of randomized placebo-controlled trials. J Gynecol Obstet Hum Reprod.

[13] Roach MK, Abramovici A, Tita AT (2013). Dose and duration of oxytocin to prevent postpartum hemorrhage: a review. Am J Perinatol.

[14] Bradley SE, Prata N, Young-Lin N, Bishai DM (2007). Cost-effectiveness of misoprostol to control postpartum hemorrhage in low-resource settings. Int J Gynaecol Obstet.

[15] Tunçalp Ö, Hofmeyr GJ, Gülmezoglu AM (2012). Prostaglandins for preventing postpartum haemorrhage. Cochrane Database Syst Rev.

[16] Su LL, Chong YS, Samuel M (2012). Carbetocin for preventing postpartum haemorrhage. Cochrane Database Syst Rev.

[17] Meshykhi LS, Nel MR, Lucas DN (2016). The role of carbetocin in the prevention and management of postpartum haemorrhage. Int J Obstet Anesth.

[18] Awoleke JO, Adeyanju BT, Adeniyi A, Aduloju OP, Olofinbiyi BA (2020). Randomised controlled trial of sublingual and rectal misoprostol in the prevention of primary postpartum haemorrhage in a resource-limited community. J Obstet Gynaecol India.

[19] (2011). Cochrane Handbook for Systematic Reviews of Interventions. https://training.cochrane.org/handbook/current?utm_medium=email&utm_source=transaction.

[20] Page MJ, McKenzie JE, Bossuyt PM (2021). The PRISMA 2020 statement: an updated guideline for reporting systematic reviews. Syst Rev.

[21] Sterne JA, Savović J, Page MJ (2019). RoB 2: a revised tool for assessing risk of bias in randomised trials. BMJ.

[22] Egger M, Davey Smith G, Schneider M, Minder C (1997). Bias in meta-analysis detected by a simple, graphical test. BMJ.

[23] Higgins JP, Thompson SG, Deeks JJ, Altman DG (2003). Measuring inconsistency in meta-analyses. BMJ.

[24] Abd El-Wahab AS, Ahmed AAK, Marai AAE (2020). The effect of carbetocin compared to rectal misoprostol in the management of blood loss during the third stage of vaginal delivery in low risk patients for postpartum hemorrhage. Al-Azhar Med J.

[25] Hetiba YA, Mahmoud MS, Oun AEM (2021). Carbetocin versus rectal misoprostol to decrease blood loss in vaginal delivery in high risk patients for postpartum hemorrhage. IJMA.

[26] Maged AM, Waly M, Fahmy RM (2020). Carbetocin versus rectal misoprostol for management of third stage of labor among women with low risk of postpartum hemorrhage. Int J Gynaecol Obstet.

[27] Abd El Aziz MA, Iraqi A, Abedi P, Jahanfar S (2018). The effect of carbetocin compared to misoprostol in management of the third stage of labor and prevention of postpartum hemorrhage: a systematic review. Syst Rev.

[28] Sallam HF, Shady NW (2018). Adjunctive sublingual misoprostol for secondary prevention of post-partum hemorrhage during cesarean delivery: double blind placebo randomized controlled trial. Int J Reprod Contracept Obstet Gynecol.

[29] Attilakos G, Psaroudakis D, Ash J (2010). Carbetocin versus oxytocin for the prevention of postpartum haemorrhage following caesarean section: the results of a double-blind randomised trial. BJOG.

[30] Ibrahim KA, Saad AS (2017). Prevention of postpartum haemorrhage in patients with severe preeclampsia using carbetocin versus misoprostol. Apollo Med.

[31] Larciprete G, Montagnoli C, Frigo M (2013). Carbetocin versus oxytocin in caesarean section with high risk of post-partum haemorrhage. J Prenat Med.

[32] Lawrie TA, Rogozińska E, Sobiesuo P, Vogel JP, Ternent L, Oladapo OT (2019). A systematic review of the cost-effectiveness of uterotonic agents for the prevention of postpartum hemorrhage. Int J Gynaecol Obstet.

[33] Matthijsse S, Andersson FL, Gargano M, Yip Sonderegger YL (2022). Cost-effectiveness analysis of carbetocin versus oxytocin for the prevention of postpartum hemorrhage following vaginal birth in the United Kingdom. J Med Econ.

[34] Vlassoff M, Diallo A, Philbin J, Kost K, Bankole A (2016). Cost-effectiveness of two interventions for the prevention of postpartum hemorrhage in Senegal. Int J Gynaecol Obstet.

[35] Jaffer D, Singh PM, Aslam A, Cahill AG, Palanisamy A, Monks DT (2022). Preventing postpartum hemorrhage after cesarean delivery: a network meta-analysis of available pharmacologic agents. Am J Obstet Gynecol.

[36] Gallos I, Williams H, Price M (2019). Uterotonic drugs to prevent postpartum haemorrhage: a network meta-analysis. Health Technol Assess.

